# FeNO may serve as a noninvasive screening tool for gastroesophageal reflux disease in children under 5 years: a retrospective study

**DOI:** 10.3389/fped.2026.1925154

**Published:** 2026-10-05

**Authors:** Fang Wang, Ying Cheng, Xiaoqin Zhou, Junhua Shu, Lin Deng, Ruxin Zhang, Yi Gan, Zhongjian Wang

**Affiliations:** Department of Pediatrics, Maternal and Child Health Hospital of Hubei Province, Tongji Medical College, Huazhong University of Science and Technology, Wuhan, China

**Keywords:** children, chronic cough, fractional exhaled nitric oxide, gastroesophageal reflux disease, ph and multiple intraluminal impedance monitoring

## Abstract

**Objective:**

To investigate the correlation between fractional exhaled nitric oxide (FeNO) measurement and pediatric gastroesophageal reflux disease (GERD), explore potential non-invasive screening indicators for pediatric GERD.

**Methods:**

A retrospective cohort of 159 children with suspected GERD was enrolled from the Department of Pediatrics, Maternal and Child Health Hospital of Hubei Province between April 2017 and August 2025. Data on 24 h ph and multiple intraluminal impedance monitoring and FeNO were systematically collected and analyzed.

**Results:**

Among 159 children, 32 (20.1%) were diagnosed with GERD. In the 101 children under 5 years, 23 (22.8%) were GERD-positive. The median FeNO in the GERD-positive group was significantly higher than that in the GERD-negative group (18.00 vs. 10.00 ppb, *P* < 0.001). Spearman analysis revealed that FeNO was significantly correlated with the number of reflux cycles (*P* = 0.012) and pH reflux cycles (*P* = 0.010). ROC analysis for FeNO predicting GERD yielded an AUC of 0.751 (*P* = 0.000), with an optimal cutoff of 17.5 ppb, a sensitivity of 52.2%, and a specificity of 87.2%. Multivariate logistic regression further confirmed FeNO as an independent risk factor for GERD in children under 5 years. However, in the 58 children aged ≥5 years, no significant difference in GERD-positive rate was observed between the FeNO_50_-positive and FeNO_50_-negative groups (*P* = 1.00).

**Conclusions:**

Elevated FeNO is independently associated with GERD in children under 5 years, showing moderate diagnostic value as a potential non-invasive screening tool, but not in those aged ≥5 years.

## 1 Introduction

Gastroesophageal reflux (GER) is defined as the retrograde flow of gastric contents into the esophagus, with or without vomiting. Chronic and recurrent GER can induce reflux esophagitis and respiratory complications including cough, wheezing, chronic sinusitis, and recurrent pneumonia, thereby impeding normal growth and development. The disease state characterized by persistent symptoms or complications secondary to GER is referred to as gastroesophageal reflux disease (GERD) (1–3). 24 h ph and multiple intraluminal impedance (pH-MII) monitoring is the most objective diagnostic method for GERD and is recognized as the gold standard (3–5). The incidence of pediatric GERD may be underestimated due to its diverse clinical manifestations, reflux symptoms often masked by extraesophageal symptoms, inability of younger children to fully express their discomfort, and the limited widespread use of 24 h pH-MII monitoring technology (6, 7).Therefore, there is an urgent need for a non-invasive, repeatable, and pathophysiologically relevant biomarker to optimize the early detection and management of GERD.

Fractional exhaled nitric oxide (FeNO) is generated by resident cells and inflammatory cells on the airway surface via the oxidation of nitric oxide synthase. It acts as a biomarker for eosinophilic airway inflammation and has been extensively applied in clinical practice for evaluating disease severity and monitoring treatment responses in bronchial asthma, chronic obstructive pulmonary disease, and chronic cough (8–10). However, recent studies have indicated that gastric acid reflux can activate NOS in the esophageal epithelium and induce airway nitric oxide release via the “esophagus-airway axis” mechanism, thereby elevating FeNO levels. This extends its classical localization to the respiratory system, rendering it a potential inflammatory signal associated with GERD (11, 12). To date, clinical studies investigating the correlation between GERD and FeNO remain limited globally. This study aims to analyze the correlation between 24 h pH-MII monitoring and FeNO measurements in children, and to explore whether FeNO can serve as a potential non-invasive screening biomarker for pediatric GERD, thereby optimizing the diagnostic workflow and treatment response monitoring for GERD.

## 2 Subjects and methods

### 2.1 Subjects

This study retrospectively analyzed the medical records of 159 children with suspected GERD who underwent 24 h pH-MII monitoring and FeNO measurement at Maternal and Child Health Hospital of Hubei Province from April 2017 to August 2025 (see Figure 1 for the case selection flowchart). Inclusion criteria: (a) presence of reflux manifestations (e.g., acid regurgitation, vomiting, throaty rales); (b) recurrent respiratory manifestations (e.g., cough, wheezing, aspiration pneumonia); (c) esophageal irritation manifestations (e.g., heartburn, chest tightness, chest pain). Eligible participants were those who met at least one of the above three criteria and simultaneously underwent 24 h pH-MII monitoring and FeNO measurement. Exclusion criteria: Patients with severe cardiovascular, respiratory, or digestive system malformations. Patients with a confirmed diagnosis of cow's milk protein allergy (CMPA) or eosinophilic esophagitis (EoE) based on prior clinical evaluation, including oral food challenge for CMPA or upper endoscopy with esophageal biopsies for EoE, were excluded from the study.

**Figure 1:**
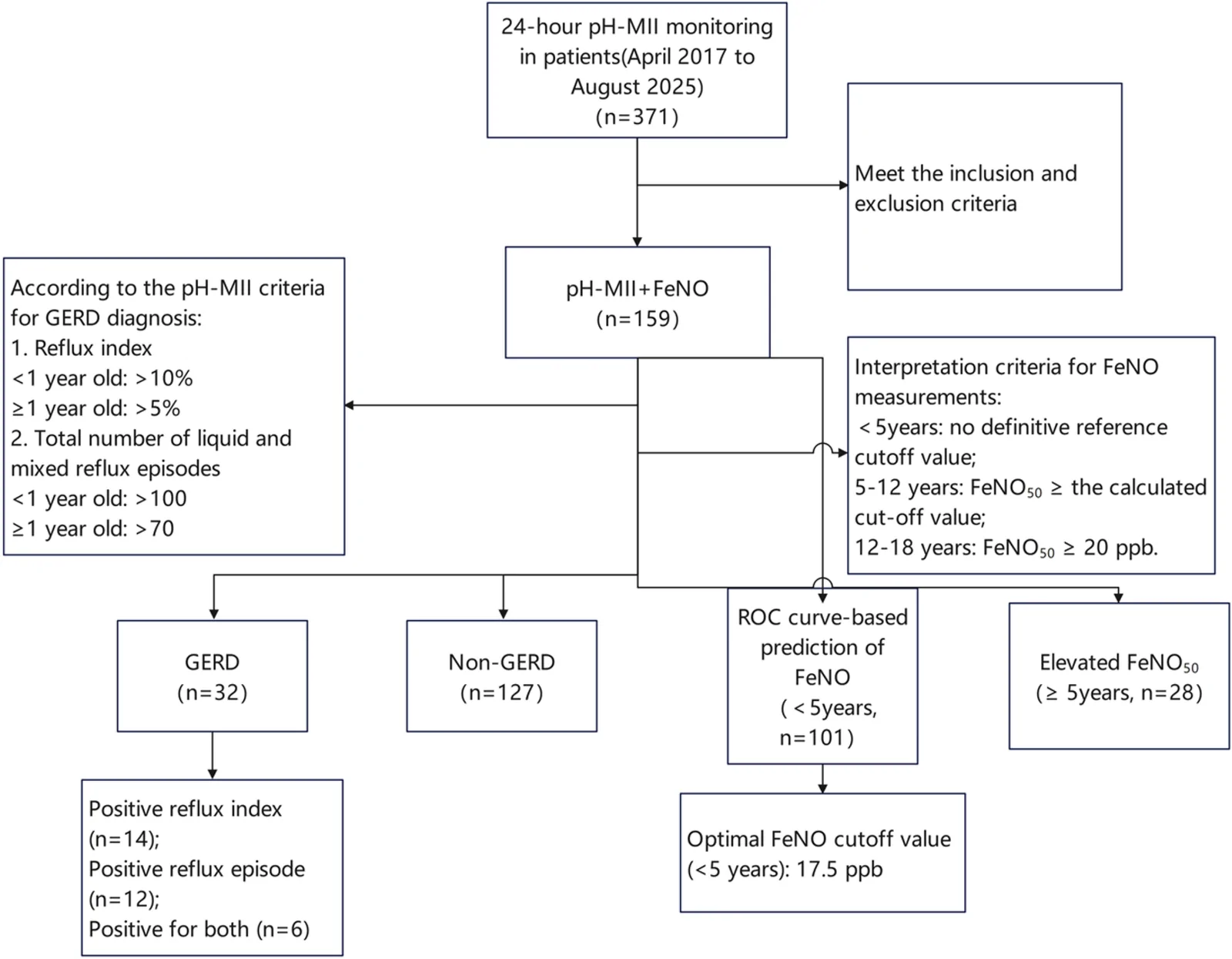
Flowchart of case screening. GERD, gastroesophageal reflux disease; FeNO, fractional exhaled nitric oxide; ROC, receiver operating characteristic.

None of the patients had received any acid-suppressing medications, including proton pump inhibitors (PPIs), histamine-2 receptor antagonists (H2RAs), or antacids, prior to or during the pH-MII monitoring and FeNO measurements. This was verified through detailed medical history interviews at the time of enrollment.

This study was approved by the Medical Ethics Committee of Maternal and Child Health Hospital of Hubei Province (Ethical Approval No. 2026-120-01) and complies with the ethical principles outlined in the Declaration of Helsinki.

### 2.2 Methods

24 h pH-MII Monitoring: 24 h pH-MII monitoring was performed using a Sandhill fixed measurement system (Sleuth, Sandhill Scientific, Inc.; Highlands Ranch, CO, USA). The probe was inserted nasally into the esophagus and calibrated with buffer solutions of pH 4.0 and 7.0. Following calibration, the probe was positioned such that the esophageal pH sensor was placed at the level of the second vertebra above the diaphragm. The variables analyzed included esophageal acid exposure, total number of reflux events, counts of acidic, weakly acidic, and alkaline reflux episodes, and the height of reflux events. Liquid reflux events were defined as a >50% decrease in impedance in at least two of the most distal impedance channels, and were classified into acidic (pH < 4), weakly acidic (pH 4–7), or weakly alkaline (pH > 7) categories. The definition of GERD was adjusted based on patient age: for infants aged <1 year, GERD was defined as a reflux index (RI) ≥10% or a total number of reflux episodes > 100; for children aged ≥1 year, the diagnostic criteria were an RI ≥5% or a total number of reflux episodes >70 (13).

FeNO_50_ Measurement: FeNO_50_ was measured using the Shangwo Naku coulometric exhaled nitric oxide analyzer (Nano Coulomb Breath Analyzer, Sunvou-CA2122, Wuxi, China), in accordance with the standardized procedures recommended by the American Thoracic Society (ATS) and the European Respiratory Society (ERS) (14). FeNO_50_ is defined as the FeNO value measured at a flow rate of 50 mL/s, which reflects nitric oxide levels in the large airways. Participants were required to fast and refrain from strenuous exercise or pulmonary function testing for 1 h prior to the measurement.

Online measurement method (applicable to children aged ≥5 years): The standardized online measurement procedure, following the ATS/ERS 2005 recommendations (14) and the Chinese Expert Consensus (2025 Edition) (15), comprised the following steps:

1. Gas inhalation: Prior to measurement, the subject performed a deep exhalation to maximize lung emptying, followed by inhalation of NO-free air (NO < 5 ppb) through a mouthpiece connected to a NO scrubber to avoid environmental NO contamination.
2. Inspiration phase: The subject held the mouthpiece and inhaled for 2–3 s until reaching or approximating total lung capacity, then immediately initiated exhalation to avoid the confounding effect of breath-holding on FeNO_50_ values.
3. Exhalation phase: A continuous exhalation was performed against resistance, with an exhalation pressure of 5–20 cmH_2_O to ensure soft palate closure and eliminate nasal NO contamination. The exhalation flow rate was maintained at 45–55 mL/s, with real-time visual biofeedback to achieve a stable NO concentration plateau.
4. Exhalation duration: Slow, steady exhalation was sustained for at least 4–6 s (≥4 s for children ≤12 years old, ≥6 s for those >12 years old). The mean value within a 3 s window during the plateau phase was used for interpretation.

Tidal breathing measurement method (applicable to infants and young children <5 years old): For younger children who could not cooperate with the online method, tidal breathing measurement was performed. First, a face mask was secured over the infant's nose and mouth while keeping the subject calm. The child inhaled air with NO < 4 ppb through an air filter, maintaining calm breathing throughout the 30–60 s process. A gas collection bag was attached to the outlet of the tidal sampler to collect exhaled gas, which was then introduced into the FeNO analyzer for detection. As the paranasal sinuses of infants are not yet fully developed, nasal NO contamination is considered negligible in this age group; however, this method is not recommended for older children due to potential interference from nasal NO.

Result interpretation: For subjects aged 12–18 years, an FeNO_50_ level ≥20 ppb is indicative of abnormality. For subjects aged 5–12 years, the FeNO_50_ cutoff value decreases by 1 ppb per year of age reduction; when the FeNO_50_ level meets or exceeds the calculated cutoff value, it is suggestive of abnormality (15–17). No definitive reference cutoff value for FeNO has been established in children under 5 years of age. The determination of FeNO abnormality requires a comprehensive assessment integrating the child's clinical manifestations, allergic history, and results of other pulmonary function tests.

### 2.3 Statistical analysis

Data were processed using SPSS 27.0 statistical software. Continuous variables were first tested for normality: those following a normal distribution were expressed as mean ± standard deviation (*x¯* ± *s*), and comparisons between two independent groups were performed using the independent-samples *t*-test; those with non-normal distributions were presented as median (interquartile range, *Q*1, *Q*3) [*M* (*Q*1, *Q*3)], and intergroup comparisons were conducted using the Mann–Whitney *U* test. Categorical data were expressed as frequency (percentage) [*n* (%)], and intergroup comparisons were performed using the Pearson's chi-square (*χ*^2^) test or Fisher's exact test (where applicable). Spearman correlation analysis was used to examine the relationship between the two variables. The optimal cutoff value was predicted using the area under the receiver operating characteristic curve (ROC curve). Risk factors were analyzed using binary logistic regression. A two-tailed *P* value <0.05 was considered statistically significant.

## 3 Results

### 3.1 Demographic and clinical characteristics

A total of 159 pediatric patients were included, with 104 males (65.4%) and 55 females (34.6%). The age range was 1 month to 12 years and 6 months, with a median age of 3 years and 1 month. Clinical presentations were as follows: cough (129 cases, 81.1%), wheezing (55 cases, 34.6%), audible phlegm rales in the throat (8 cases, 5.0%), vomiting (4 cases, 2.5%), and chest tightness (2 cases, 1.3%). A total of 62 patients (39.0%) had a history of allergies. Comorbidities or pre-existing conditions included 4 cases of atrial septal defect, 1 case of functional dysphagia, 1 case of congenital diaphragmatic eventration, 1 case of congenital myasthenia gravis, and 1 case of obliterative bronchiolitis.

### 3.2 Comparison between the GERD-positive group and GERD-negative group

Among the 159 pediatric patients, 32 (20.1%) met the diagnostic criteria for GERD based on 24 h pH-MII monitoring. Specifically, 14 cases (43.8%) had a positive reflux index, 12 cases (37.5%) had a positive number of reflux episodes, and 6 cases (18.8%) were positive for both. Subgroup analysis by age showed that 8 patients (25.0%) were under 1 year old, 24 patients (75.0%) were aged 1 year or older, with a median age of 1 year and 2 months. Significant differences were observed between children aged <1 year and those aged ≥1 year in number of reflux episodes, number of pH reflux cycles, maximum reflux duration, weakly acidic mixed reflux, and total reflux (mixed + gas) (all *P* < 0.05). However, no significant differences were found between the two age groups in GERD positivity rate, reflux index, and acid reflux (as shown in the Table 1).

**Table 1:** Comparison of reflux in infants and children.

Variable	Age < 1 year (*n* = 46)	Age ≥ 1 year (*n* = 113)	*P*-value
Sex (Male)	35 (76.09%)	69 (61.06%)	0.097
GERD-Positive	8 (17.39%)	24 (21.24%)	0.667
Reflux index	1.95 (0.75,5.38)	1.10 (0.70,1.80)	0.063
Number of reflux episodes	39.00 (10.00,68.50)	5.00 (0.00,32.50)	<0.01
Number of PH reflux cycles	20.00 (8.50,43.25)	10.00 (6.00,19.00)	0.012
Maximum reflux duration	10.00 (2.73,25.13)	2.60 (1.60,5.00)	<0.01
Total (acid) reflux	25.00 (12.75,41.25)	17.00 (12.00,29.00)	0.075
Liquid (acid) reflux	6.50 (1.75,18.00)	4.00 (2.00,8.00）	0.076
Gas (acid) reflux	2.00 (0.00,5.00)	1.00 (0.00,3.00)	0.061
Mixed (acid) reflux	14.00 (6.00,28.25)	11.00 (7.00,16.00)	0.187
Liquid (weak acid) reflux	11.50 (5.00,25.50)	8.00 (5.00,17.00)	0.193
Gas (weak acid) reflux	26.00 (12.00,40.00)	20.00 (10.00,35.00)	0.534
Mixed (weak acid) reflux	22.00 (11.00,33.25)	11.00 (6.00,20.00)	<0.01
Total liquid reflux	27.50 (9.00,41.00)	20.00 (12.00,33.00)	0.282
Total mixed reflux	45.00 (27.75,57.75)	30.00 (17.00,43.00)	<0.01
Total gas reflux	33.00 (20.00,47.25)	40.00 (24.00,80.00)	0.033

GERD, gastroesophageal reflux disease.

Among the 32 GERD children, 15 (46.9%) were exclusively breastfed; 10 (31.2%) were delivered vaginally and 20 (62.5%) by cesarean section; 13 (40.6%) had a history of allergies; and 12 (37.5%) had a history of recurrent respiratory infections. No statistically significant differences were observed between the GERD-positive group and GERD-negative group regarding baseline characteristics including age, gender, birth history, feeding history, allergy history, and history of recurrent respiratory tract infections (all *P* > 0.05) (see Table 2).

**Table 2:** Comparison of baseline characteristics between the GERD-positive group and GERD-negative group.

Variable	GERD-positive group (*n* = 32)	GERD-negative group (*n* = 127)	*t*/*X*^2^ value	*P*-value
Age				
<1 y	8 (25.0%)	38 (29.9%)	0.301	0.667
≥1 y	24 (75.0%)	89 (70.1%)		
Sex				
Male	18 (56.3%)	86 (67.7%)	1.485	0.298
Female	14 (43.7%)	41 (32.3%)		
Birth history				
Full-term birth	29 (90.6%)	104 (81.9%)	0.018	1.000
Preterm birth	2 (6.3%)	8 (6.3%)		
Mode of delivery				
Vaginal delivery	10 (31.2%)	48 (37.8%)	1.031	0.404
Cesarean section	20 (62.5%)	62 (48.8%)		
Feeding mode				
Breastfeeding	15 (46.9%)	56 (44.1%)	0.041	1.000
Formula/mixed feeding	16 (50.0%)	55 (43.3%)		
Allergy history				
Allergy-related symptoms	13 (40.6%)	57 (44.9%)	1.306	0.268
Positive for food/drug/inhalant allergens	8 (25.0%)	39 (30.7%)		
**History of recurrent respiratory tract infections**	12 (37.5%)	27 (21.3%)	3.642	0.068

GERD, gastroesophageal reflux disease.

### 3.3 Comparison of the association between FeNO and GERD across different age groups

Among the 101 children under 5 years of age, 23 (22.8%) were GERD-positive. The median FeNO level in the GERD-positive group was 18.00 (10.00, 26.00) ppb, which was significantly higher than that in the GERD-negative group [10.00 (6.00, 13.00) ppb, *P* < 0.001]. Children in the GERD-positive group also exhibited significantly higher values in reflux index (*P* < 0.001), number of reflux episodes (*P* < 0.001), number of pH reflux cycles (*P* < 0.001), maximum reflux duration (*P* < 0.001), acid reflux (*P* < 0.001), and liquid reflux (*P* = 0.004) compared with the GERD-negative group (Table 3). Spearman correlation analysis between FeNO and reflux parameters revealed that FeNO was significantly correlated with the number of reflux cycles (*P* = 0.012) and the number of pH reflux cycles (*P* = 0.010) (Table 4).

**Table 3:** Comparison of reflux status and FeNO between the GERD-positive and negative groups in children (<5 years old).

Variable	GERD-positive group (*n* = 23)	GERD-negative group (*n* = 78)	*Z* value	*P*-value
FeNO	18.00 (10.00,26.00)	10.00 (6.00,13.00)	3.660	<0.001
Reflux index	9.70 (6.00,23.10)	1.10 (0.60,2.00)	6.301	<0.001
Number of reflux episodes	67.00 (18.00,87.00)	17.00 (0.00,42.00)	3.807	<0.001
Boix-Ochoa score	65.91 (39.96,149.31)	8.38 (4.67,14.22)	5.831	<0.001
Number of PH reflux cycles	139.00 (58.00,206.00)	16.00 (8.00,37.50)	5.543	<0.001
Maximum reflux duration	36.00 (6.20,168.10)	4.00 (1.85,10.00)	4.431	<0.001
Liquid (acid) reflux	18.00 (8.00,30.00)	4.00 (1.00,8.00)	4.801	<0.001
Gas (acid) reflux	4.00 (1.00,10.00)	1.00 (0.00,3.00)	2.796	0.005
Mixed (acid) reflux	21.00 (7.00,50.00)	10.50 (4.50,17.00)	3.249	0.001
Total (acid) reflux	36.00 (27.00,88.00)	17.00 (8.75,27.25)	4.476	<0.001
Total liquid reflux	32.00 (18.00,52.00)	20.00 (9.00,34.00)	2.896	0.004
Total mixed reflux	48.00 (25.00,63.00)	31.00 (18.00,47.00)	1.660	0.097

GERD, gastroesophageal reflux disease; FeNO, fractional exhaled nitric oxide.

**Table 4:** FeNO and reflux parameter spearman correlation analysis in children (<5 years old).

Variable	Spearman correlation coefficient	*P*-value
Reflux index	0.191	0.056
Number of reflux episodes	0.159	0.113
Boix-Ochoa score	0.142	0.157
Number of reflux cycles	0.248	0.012
Number of PH reflux cycles	0.255	0.010
Maximum reflux duration	0.060	0.552
Liquid (acid) reflux	0.185	0.064
Total (acid) reflux	0.083	0.406

FeNO, fractional exhaled nitric oxide.

When applying the area under the ROC curve to predict GERD in children under 5 years of age, the area under the curve of FeNO was 0.751 (*P* = 0.000). The optimal cutoff value was calculated to be 17.5 ppb, with a sensitivity of 52.2% and a specificity of 87.2% (Table 5 and Figure 2).

**Table 5:** ROC curve analysis of GERD in children (<5 years old).

Variable	Area	95% CI	*P* Value	Sensitivity (%)	Speciality (%)
FeNO	0.751	0.644–0.859	0.000	52.2	87.2

GERD, gastroesophageal reflux disease; FeNO, fractional exhaled nitric oxide; ROC, receiver operating characteristic.

**Figure 2:**
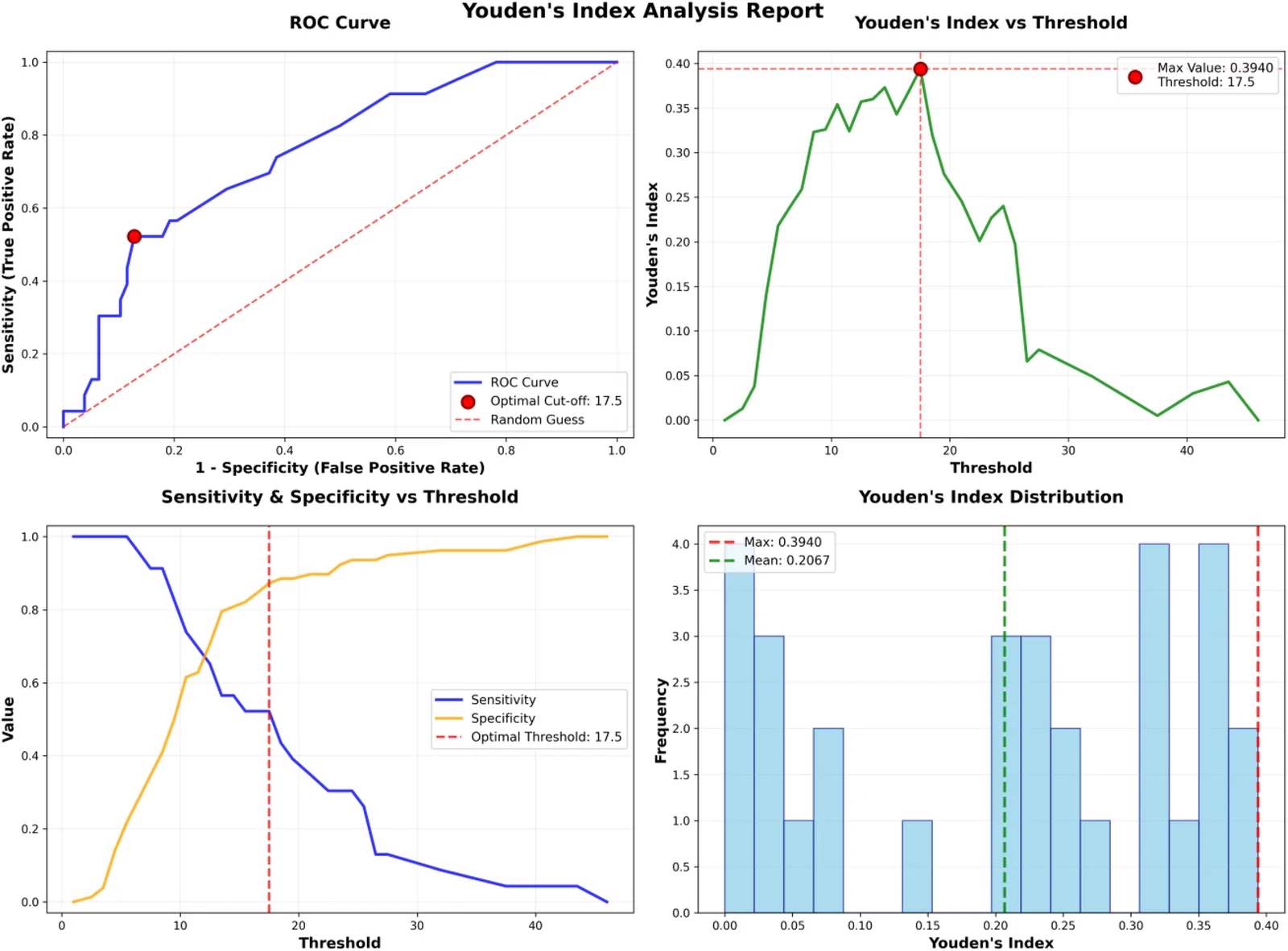
ROC curve and Youden's index analysis for FeNO in children (<5 years old). FeNO, fractional exhaled nitric oxide; ROC, receiver operating characteristic.

Multivariate logistic regression analyses were conducted to examine the associations between age, sex, mode of delivery, birth history, feeding pattern, allergy history, FeNO level and GERD. Multivariate logistic regression analysis showed that FeNO was an independent risk factor for GERD in children under 5 years of age (Table 6).

**Table 6:** Multivariate logistic regression analysis of risk factors associated with GERD in children (<5 years old).

Variable	*B*	SE	Wald	Exp (*B*)	95% CI	*P*
Age	−0.012	0.018	0.399	0.989	0.954–1.025	0.527
Sex	−1.001	0.575	3.028	0.368	0.119–1.135	0.082
Mode of delivery	−0.849	0.597	2.025	0.428	0.133–1.378	0.155
Birth history	0.252	0.903	0.078	1.286	0.219–7.555	0.780
Feeding mode	0.147	0.545	0.073	1.159	0.398–3.372	0.787
Allergy history	−0.530	0.597	0.788	0.589	0.183–1.897	0.375
FeNO	0.084	0.029	8.347	1.087	1.027–1.150	0.004

GERD, gastroesophageal reflux disease; FeNO, fractional exhaled nitric oxide.

In the present study, 58 children aged 5 years and older were included, among whom 28 (48.3%) were FeNO_50_-positive and 9 (15.5%) were GERD-positive. There was no significant difference in the GERD-positive rate between the FeNO_50_-positive group and the FeNO_50_-negative group (*P* = 1.00) (Table 7).

**Table 7:** Comparison of GERD between FeNO_50_-positive group and negative group in children (≥5 years old).

Group	FeNO_50_-positive group (*n* = 28)	FeNO_50_-negative group (*n* = 30)	*P*-value
GERD-positive	4 (6.90%)	5 (8.62%)	1.00
GERD-negative	24 (41.38%)	25 (43.10%)	

GERD, gastroesophageal reflux disease; FeNO, fractional exhaled nitric oxide.

## 4 Discussion

With the socio-economic development and improvement of living standards, the incidence of GER has been increasing year by year. GERD is a prevalent gastrointestinal motility disorder, and its symptoms and complications may stem from multifactorial mechanisms. Specifically, gastric acid reflux stimulates esophageal squamous epithelial cells to secrete chemokines, which recruit inflammatory cells and ultimately result in esophageal mucosal injury (18). Studies have demonstrated that acidic aspiration (including gastric acid and bile salts) can induce interleukin-6 (IL-6)- and interleukin-8 (IL-8)-mediated neutrophil infiltration into the lungs, thereby causing direct or indirect damage to airway epithelial cells (19). Pepsin, by contrast, reduces the levels of two laryngeal protective proteins—carbonic anhydrase III (CAIII) and Sep70—resulting in laryngeal mucosal injury (20). Furthermore, the proteolytic activity of pepsin combined with acid-induced damage may exacerbate cellular and tissue injury. Specifically, studies have shown that pepsin exerts more severe damage to rabbit esophageal tissue in an acidic environment than acid alone (21). FeNO testing is a non-invasive diagnostic method that is widely utilized as a biomarker for monitoring eosinophilic airway inflammation and guiding personalized treatment of asthma. However, research on its association with GERD remains scarce.

Through a retrospective analysis of 159 children with suspected GERD, this study systematically explored the association between FeNO and GERD in the pediatric population for the first time. The findings of this study revealed that in children under 5 years of age, the median level of FeNO was higher in the group with positive GERD compared to the group with negative GERD. FeNO was significantly correlated with the number of reflux cycles and the number of pH reflux cycles. More importantly, we determined the optimal FeNO cutoff value for predicting GERD in children under 5 years using ROC curve analysis to be 17.5 ppb, with a sensitivity of 52.2% and a specificity of 87.2%. Multivariate logistic regression analysis showed that FeNO is an independent risk factor for GERD in children under 5 years of age. These findings indicate that elevated FeNO is not only a classic biomarker for airway eosinophilic inflammation but may also be closely associated with the severity of GERD in children. This suggests that FeNO may serve as a potential non-invasive biomarker for evaluating GERD-related airway inflammation and reflux burden in children under 5 years. We also provided reference thresholds for FeNO measured by tidal breathing method in children under 5 years of age suspected of having GERD.

The mechanism underlying elevated FeNO in pediatric patients with GERD is multifactorial, potentially involving interactions within the “esophagus-airwa*y* axis.” First, reflux and microaspiration of gastric contents—especially gastric acid and pepsin—can directly damage the airway epithelium (21). Second, refluxate can indirectly induce airway inflammation via neural reflex mechanisms (22, 23). Furthermore, a study focusing on gastroesophageal reflux-related cough (GERC) demonstrated that GERC patients with elevated alveolar nitric oxide (CaNO) exhibited significantly higher levels of pepsin, IL-8, matrix metalloproteinase-9 (MMP-9), and mucin in induced sputum compared to the CaNO-normal group. This finding provides direct evidence that microaspiration of gastric contents induces distal airway inflammation and nitric oxide production (24). A recently published study in the journal Life establishes a more direct pathophysiological link between FeNO and GERD. The study demonstrated that 70% of preschool children with severe wheezing had comorbid GERD, and the proportion of lipid-laden macrophages (LLMs) in their bronchoalveolar lavage fluid was significantly higher than that in children without GERD. More importantly, the proportion of LLMs was positively correlated with multiple reflux characteristics as well as symptom indices, and wheezing episodes were significantly reduced following anti-reflux therapy (25). This study proposes that LLMs can serve as a surrogate marker for reflux-related microaspiration. This finding is highly consistent with the observation in our study that FeNO was significantly correlated with the number of reflux cycles and the number of pH reflux cycles. We hypothesize that in children under 5 years of age, higher FeNO levels suggest more frequent microaspiration of refluxate, triggering airway epithelial injury and subsequent inflammatory cascades, which in turn leads to elevated FeNO levels via the activation of inducible nitric oxide synthase. This “reflux–microaspiration–airway inflammation–FeNO elevation” pathway provides robust support for FeNO as a biomarker of GERD severity and airway involvement.

In the present study, the tidal breathing FeNO level in the GERD-positive group of children under 5 years of age (18.00 ppb) was significantly higher than that in the GERD-negative group (10.00 ppb), suggesting a marked elevation of FeNO levels in children with GERD. Currently, there is no unified reference standard for tidal breathing FeNO levels in healthy children aged 1–5 years. van der Heijden et al. (26) reported a geometric mean FeNO of 7.1 ppb (95% CI 2.8–11.5 ppb) with a 95th percentile of 22.6 ppb in 51 healthy Dutch children aged 1–5 years using off-line tidal breathing. The median FeNO in the GERD-negative group of the present study was 10.00 (6.00, 13.00) ppb, which was generally consistent with this reference range. Although the diagnostic threshold of 17.5 ppb determined in this study was lower than the 95th percentile reported by van der Heijden et al. (26) (22.6 ppb), it was significantly higher than the geometric mean level in healthy children, suggesting that GERD itself may induce a mild elevation of FeNO, with the degree falling between the upper limit of the normal range and typical eosinophilic airway inflammation. Gabriele et al. (27) reported a geometric mean tidal breathing FeNO of 10.4 ppb (95% CI: 9.1–12.0 ppb) in 100 healthy infants aged 1.1–7.7 months, and Meyts et al. (28) reported a geometric mean tidal breathing FeNO of 13 ppb (95% CI: 11.8–13.7 ppb) in healthy children aged 3–6 years. The FeNO levels in the GERD-negative group of this study were similar to those in the above two studies, whereas the median FeNO of 18.00 ppb in the GERD-positive group was notably higher than the geometric means of these healthy children, further supporting the objective existence of reflux-related FeNO elevation. In the present study, multivariate logistic regression analysis showed that FeNO was an independent risk factor for GERD in children under 5 years of age, further confirming the independent association between elevated FeNO and GERD. However, in the subgroup of children aged 5 years and older, there was no significant difference in the GERD-positive rate between the FeNO_50_-positive and FeNO_50_-negative groups, suggesting that the predictive value of FeNO for GERD may be limited to infants and young children under 5 years of age using tidal breathing. This age-dependent difference may be attributable to the fact that older children undergo online FeNO_50_ measurement, which differs fundamentally from tidal breathing in terms of exhalation pattern, soft palate closure, and control of nasal NO contamination (15), and the results of the two methods are not interchangeable. Zhang et al. (24) also found in adult patients with GERC that CaNO, rather than FeNO_50_, was significantly correlated with reflux parameters, indicating that different NO parameters possess distinct clinical significance in the diagnosis of GERD. Future studies should further explore the standardization of tidal breathing FeNO measurement protocols in infants and young children and establish age-specific diagnostic thresholds, so as to fully harness the potential of FeNO as a non-invasive screening tool for GERD.

The findings of the present study hold notable clinical translational value. First, FeNO, as a non-invasive, convenient, and reproducible detection modality, can serve as an initial screening tool for children under 5 years of age with suspected GERD. Given the diverse and often atypical clinical manifestations of pediatric GERD—particularly when respiratory symptoms (e.g., chronic cough, wheezing) are predominant—FeNO testing may facilitate the identification of children who require prioritized 24 h pH-MII monitoring. Second, FeNO levels may correlate with the severity of GERD. This implies that FeNO could serve as a biomarker for monitoring the therapeutic efficacy of anti-reflux treatments, such as proton pump inhibitors. While the present study did not assess treatment response, it provides a rationale for future investigations into FeNO as a therapeutic monitoring biomarker. Finally, the study results show that when FeNO levels measured by tidal breathing in children under 5 years old exceed 17.5 ppb, the specificity for diagnosing GERD is 87.2%, significantly reducing the misdiagnosis rate of GERD.

The present study has the following limitations. First, as a retrospective study, it is inherently prone to selection bias and information bias. The relatively small sample size and single-center recruitment limit the generalizability of the findings. Second, follow-up data on FeNO levels after anti-reflux therapy were not incorporated, precluding an evaluation of the association between dynamic changes in FeNO and clinical outcomes. Additionally, owing to incomplete statistical data on eosinophil counts, this variable was temporarily excluded from the analysis in the present study, which is truly regrettable. Finally, the absence of systematic endoscopic evaluation with esophageal biopsies in all participants, and the lack of a standardized cow's milk elimination trial prior to enrollment, may have led to the inadvertent inclusion of patients with unrecognized CMPA or EoE.

Future research should prioritize multicenter, prospective, large-scale cohort studies to validate the findings of the present study. Further investigations are warranted to explore the value of FeNO and its subcomponents (e.g., CaNO, FeNO_200_) in the diagnosis, subtyping, and therapeutic response assessment of GERD. Additionally, conducting randomized controlled trials to evaluate whether FeNO-based therapeutic strategies (e.g., FeNO-guided anti-inflammatory therapy) can improve the prognosis of pediatric GERD patients will be a critical direction for future research.

## Data Availability

The raw data supporting the conclusions of this article will be made available by the authors, without undue reservation.
